# Prevalence and risk factors for lymph node metastasis in duodenal neuroendocrine tumors: a systematic review and meta-analysis

**DOI:** 10.1007/s00535-025-02247-7

**Published:** 2025-04-03

**Authors:** Yohei Ogata, Waku Hatta, Takeshi Kanno, Yutaka Hatayama, Masahiro Saito, Xiaoyi Jin, Tomoyuki Koike, Akira Imatani, Yuhong Yuan, Atsushi Masamune

**Affiliations:** 1 Division of Gastroenterology, Tohoku University Graduate School of Medicine, 1-1 Seiryo-machi, Aoba-ku, Sendai, Miyagi 980-8574 Japan; 2 Department of Medicine, London Health Science Centre, Western University, 800 Commissioners Road East, London, ON N6A 5W9 Canada

**Keywords:** Duodenal neuroendocrine tumors, Lymph node metastasis, Pathological risk factors

## Abstract

**Background:**

Although the status of lymph node metastasis (LNM) is crucial in determining treatment strategy for duodenal neuroendocrine tumors (D-NETs), robust evidence for their potential LNM risk remains lacking. This systematic review aimed to summarize the prevalence and risk factors of LNM in D-NETs.

**Methods:**

This systematic review of electronic databases identified eligible case–control and cohort studies for D-NET resected either endoscopically or surgically, published from 1990 to 2023. The primary outcome was the pooled prevalence of LNM in D-NETs. Secondary outcomes included the pooled prevalence of LNM according to tumor location and functionality, as well as identifying pathological risk factors for LNM. Meta-analysis was performed.

**Results:**

We identified 36 studies that involved 1,396 patients with D-NETs, including 326 with LNM. The pooled prevalence of LNM in D-NETs was 22.7% (95% confidence interval [CI] 17.3–29.2%). The prevalence was high in ampullary/peri-ampullary D-NETs and functional D-NETs (46.8 and 53.3%, respectively), whereas it was low in non-functional, non-ampullary D-NETs (NAD-NETs) (9.5%). Pathological risk factors for LNM in NAD-NETs included tumor size > 10 mm (odds ratio [OR] 7.31 [95% CI 3.28–16.31]), tumor invasion into the muscularis propria or deeper (OR 7.79 [3.65–16.61]), lymphovascular invasion (OR 5.67 [2.29–14.06]), and World Health Organization grading of G2 (OR 2.47 [1.03–5.92]).

**Conclusion:**

Approximately one-fourth of the patients with D-NETs had LNM. Endoscopic resection might be acceptable for non-functional NAD-NETs with diameters of 10 mm or less, but additional surgical resection with lymphadenectomy may be recommended for cases exhibiting pathological risk factors.

**Supplementary Information:**

The online version contains supplementary material available at 10.1007/s00535-025-02247-7.

## Introduction

Duodenal neuroendocrine tumors (D-NETs) are relatively rare with an incidence of 0.19 per 100,000, accounting for only 2–4% of all gastrointestinal neuroendocrine tumors (NETs) and 1–3% of primary duodenal tumors [1–3]. However, the incidence of D-NETs is rising, possibly due to the increased use of esophagogastroduodenoscopy [4].

With the development of the endoscopic resection (ER) technique, ER can achieve R0 resection even for D-NETs with deep submucosal or muscularis invasion [5, 6]. However, the therapeutic approach for D-NETs varies based on their features and the risk of lymph node metastasis (LNM). D-NETs are categorized into ampullary/peri-ampullary D-NETs (AD-NETs) and non-ampullary D-NETs (NAD-NETs), showing different clinical, histologic, and immunohistochemical features [7–9]. Pancreatoduodenectomy with lymphadenectomy is recommended for AD-NETs [10, 11], as they exhibit more aggressive biological behavior than that of NAD-NETs. In addition to the location, functional or non-functional tumors are important factors in deciding the treatment strategy because functional D-NETs, such as gastrinoma and somatostatinoma, have a higher metastatic potential [12]. ER is acceptable only for non-functional NAD-NETs. However, evidence of the differences in aggressiveness and actual prevalence of LNM across the types of D-NETs is lacking due to their rarity. The pathological risk factors for LNM, such as lymphovascular invasion (LVI) and World Health Organization (WHO) grading, are crucial to determine the indication for additional surgical resection (SR) with lymphadenectomy after ER, as treatment selection is recommended based on the presence or absence of LNM in the guidelines [10]. However, the risk factors have not been fully understood. Thus, we conducted a systematic review and meta-analysis to investigate the prevalence of and risk factors for LNM in D-NETs.

## Methods

### Search strategy and study selection

This systematic review was conducted in accordance with the Preferred Reporting Items for Systematic Reviews and Meta-Analyses (PRISMA) statement (Supplementary Appendix 1) [13], and the protocol was registered in the Prospective Register of Systematic Reviews (PROSPERO registration number: CRD42020198582, https://www.crd.york.ac.uk/prospero/). We searched the databases MEDLINE, Embase, Cochrane Central Register of Controlled Trials (CENTRAL), and Cochrane Database of Systematic Reviews (CDSR) (via OvidSP) for eligible studies that were published from 1990 to June 2023. We excluded conference proceedings, non-English-language studies, and animal studies. An experienced medical information specialist (YY), with input from the study’s investigators, designed and conducted the search strategy. Controlled vocabulary as well as keywords were used to identify studies concerning gastrointestinal NETs. Supplementary Appendix 2 details the complete search strategy.

### Study selection

We included case–control and cohort studies according to the following criteria: (i) cases with D-NETs resected either endoscopically or surgically; (ii) a minimum of 10 cases; (iii) assessment of LNM or its risk factors. The following studies were excluded: (i) published in languages other than English; (ii) case series, case reports, review articles, letters to the editor, comments, editorials, and conference proceedings; (iii) involving only patients with neuroendocrine carcinomas.

### Definitions

Diagnostic criteria for D-NETs were pathologically diagnosed NETs in the duodenum. We defined LNM as the pathological confirmation of LNM in SR cases with lymphadenectomy. Meanwhile, LNM in cases undergoing ER or local resection was defined as clinical LNM, assessed using methods such as radiological examinations before treatment and during the follow-up period. Cases that received additional SR after ER were categorized as SR cases, whereas those that underwent SR for LNM detected during follow-up after ER were classified as ER cases. Regarding functionality, the functional D-NETs included gastrinoma, somatostatinoma, gangliocytic paraganglioma (composite gangliocytoma/neuroma and NETs), glucagonoma, and insulinoma. In addition, cases with Zollinger–Ellison syndrome or von Recklinghausen’s disease were also considered to have functional D-NETs.

### Data extraction

Two reviewers (YO and WH) independently screened all titles and abstracts to identify studies meeting the inclusion criteria. After this initial screening, the full texts of relevant studies were reviewed to extract the following data: author information, country of origin, publication year, study design, study population characteristics, cohort size, treatment approach, tumor location (ampullary/peri-ampullary or non-ampullary), tumor functionality, LNM frequency, pathological factors (including tumor size, depth, WHO grading, and LVI), prognostic outcomes, and follow-up durations. A third reviewer (TK) was consulted to resolve any discrepancies between the reviewers.

### Outcomes measures

Our primary outcome was the pooled prevalence of LNM in D-NETs. The secondary outcomes included the pooled prevalence of LNM according to tumor location (AD-NETs and NAD-NETs) and tumor functionality (functional and non-functional D-NETs), as well as the identification of pathological risk factors for LNM in non-functional NAD-NETs and overall D-NETs. The following pathological risk factors were investigated: tumor size > 10 mm, tumor invasion into the muscularis propria (MP) or deeper, WHO grading of G2 or G3, and the presence of LVI.

### Quality assessment and risk of bias

Two reviewers (YO and WH) independently assessed the risk of bias in individual studies using the Joanna Briggs Institute (JBI) Critical Appraisal Tools for prevalence studies [14]. The risk level was categorized as high (≤ 49%), moderate (50–69%), and low (≥ 70%) based on the percentage of “yes” responses to the nine questions. A third reviewer (TK) was consulted to discuss and resolve any discrepancies between the two reviewers through consensus.

### Statistical analysis

We calculated the pooled proportion and its 95% confidence interval (CI) using the Freeman–Tukey double arcsine method and the inverse variance method. Additionally, we used the Mantel–Haenszel random effects model to estimate the pooled odds ratio (OR) with a 95% CI for the association between risk factors and LNM [15]. The OR can for the risk factor can be calculated if at least two studies reported the data. We utilized the inverse variance method to pool the ratios. A random effects model was applied in all meta-analyses due to the expected clinical heterogeneity between studies [16].

Statistical heterogeneity between studies was assessed using Cochran’s Q test, with a *p* value < 0.10 indicating statistical significance. Since there are no specific tests to assess heterogeneity in proportional meta-analysis, high inconsistency index (*I*^2^) values are expected [17], therefore, although *I*^2^ values were calculated for pooled prevalence, the results were interpreted conservatively. Heterogeneity for comparative outcome data was quantified with the *I*^2^ statistics, where values of < 30%, 30–59%, 60–75%, and > 75% indicated low, moderate, substantial, and considerable heterogeneity, respectively [18]. Further, we also conducted subgroup analyses to investigate the sources of heterogeneity between studies, considering a *p* value for the difference between subgroups (*p*_interaction_) of < 0.10 as statistically significant. Analyses included geographic areas (Asia, Europe, and North America) and treatment methods (ER and SR).

Tests to assess publication bias and funnel plots were developed in the context of comparative data for the pooled proportion meta-analyses. Currently, no test is recommended for assessing publication bias in the pooled proportion meta-analyses. Therefore, for pooled prevalence, while funnel plots are presented, we assessed publication bias qualitatively, as suggested [17]. Publication bias for comparative outcome data was assessed by visual inspection of the funnel plots and quantitatively evaluated using Egger’s test [19, 20], which was performed when ≥ 10 studies were available in the meta-analysis, congruent with previous recommendations [21]. A *p* value ≤ 0.10 in this test indicated publication bias. Statistical analyses were performed using R version 4.2.1 (R Foundation).

## Results

### Search and selection of studies

**Fig. 1:**
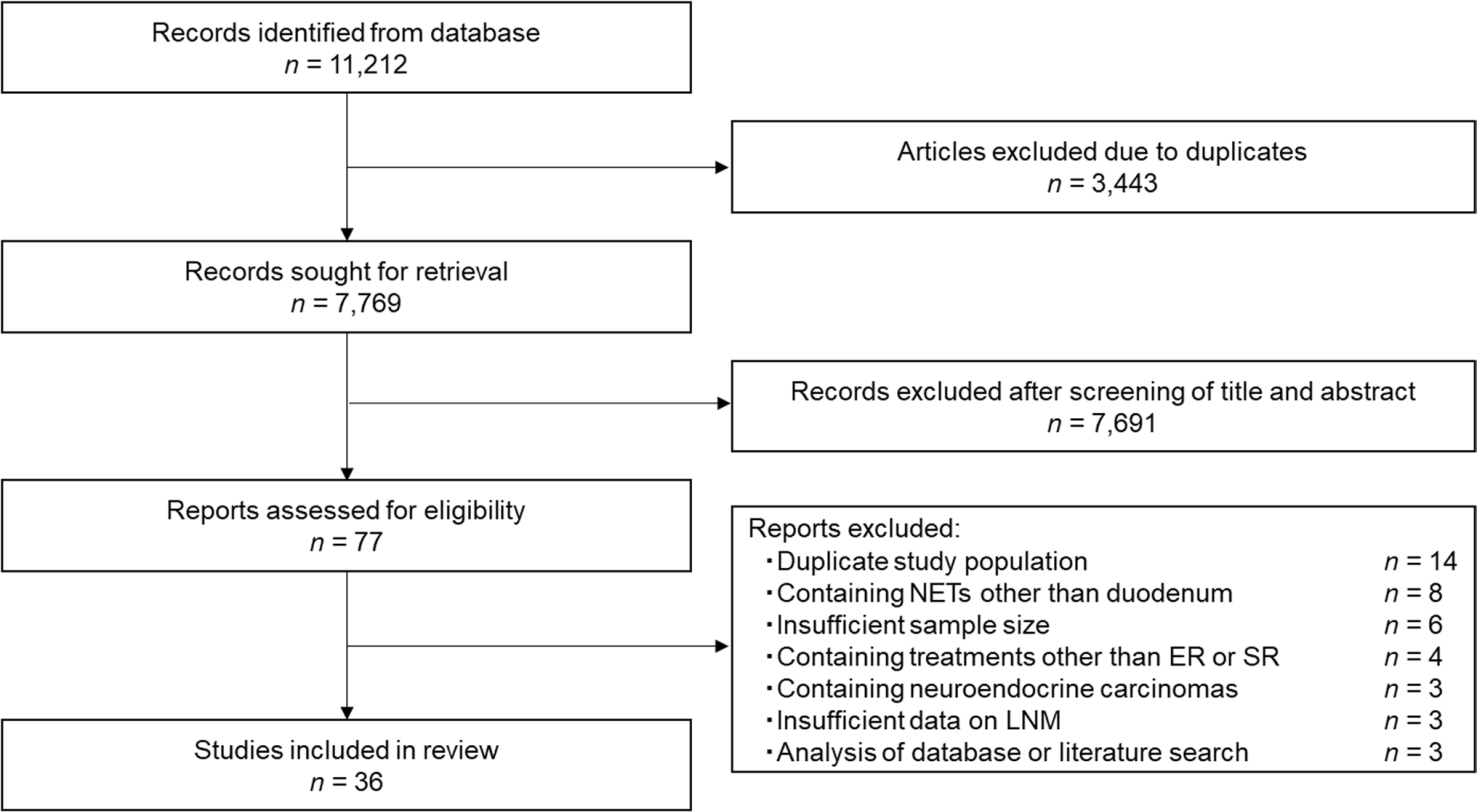
PRISMA flow diagram of studies identified in the systematic review. *ER* endoscopic resection, *LNM* lymph node metastasis, *NETs* neuroendocrine tumors, *PRISMA* Preferred Reporting Items for Systematic Reviews and Meta-Analyses, *SR* surgical resection

The search strategy identified 11,212 articles on gastrointestinal NETs from the database. A total of 7,769 titles and abstracts were screened after excluding 3,443 articles due to duplicates. Subsequently, we retrieved and assessed 78 full-text articles that were potentially eligible. Of these, 36 articles [5, 22–56] were included in the final analysis. Figure 1 presents the PRISMA flow diagram of the studies identified in this analysis.

### Characteristics of the included studies

Table 1 and Supplementary Table 1 show the baseline characteristics of all included studies, consisting of nine multi-center studies [27, 29, 34, 39, 41, 44, 46, 47, 53] and 27 single-center studies [5, 22–26, 28, 30–33, 35–38, 40, 42, 43, 45, 48–52, 54–56]. Of the studies, 14 originated from Asia [5, 30, 32, 34, 40, 41, 45, 47–49, 52, 54–56], nine from Europe [25, 27, 29, 33, 38, 39, 44, 50, 51], and 12 from North America [22–24, 26, 28, 35–37, 42, 43, 46, 53]. Based on the JBI Critical Appraisal Tools for study quality assessment, 26 and 10 studies were classified as being at low and moderate risks, respectively (Supplementary Table 2). No studies were classified as high risk.

**Table 1:** Main characteristics of the included studies in the analysis

Authors	Year	Country	Enrollment time period	Type of treatment (*n*)	No. of patients	LNM in SR cases	LNM in all cases
Delcore Jr et al.	1990	US	1960–1990	SR (15)	15	10	10
Burke et al.	1990	US	1970–1986	SR (67)	67	13	13
Sugg et al.	1993	US	1989–	SR (27)	27	14	14
Kisker et al.	1998	Germany	1987–1996	SR (10)	10	5	5
Bornstein-Quevedo et al.	2001	Mexico	1980–2000	SR (13)	13	5	5
Witzigmann et al.	2002	Germany	1992–2001	ER (2), SR (9)	11	1	1
Mullen JT et al	2005	US	1969–2004	ER (6), SR (18)	24	7	7
Bartsch et al.	2012	Germany	1990–2011	SR (26)	26	16	16
Min et al.	2013	Korea	1996–2009	ER (11)	11	NA	0
Waisberg et al.	2013	Brazil	1993–2011	ER (15), SR (5)	20	1	1
Kim et al.	2013	Korea	2001–2011	ER (12), SR (1)	13	0	0
Chopin-Laly et al.	2013	France	1991–2007	ER (NA), SR (NA)	34	NA	11
Kim et al.	2014	Korea	2006–2011	ER (41)	41	NA	0
Untch et al.	2014	US	1983–2011	ER (12), SR (53)	65	16	16
Shroff et al.	2015	US	2001–2011	ER (20), SR (10)	30	0	0
Sheikh et al.	2016	US	2005–2014	SR (18)	18	7	6
Rosentraeger et al.	2016	Germany	1984–2008	ER (10), SR (25)^a^	41	16	16
Gincul et al.	2016	France	1996–2003	ER (26), SR (3)^b^	29	2	3
Iwasaki et al.	2017	Japan	2000–2015	SR (13)	13	7	7
Hatta et al.	2017	Japan	1992–2013	ER (35), SR (14)	49	6	7
Dogeas et al.	2017	US	1996–2012	ER (38), SR (63)	101	27	27
Weatherall et al.	2017	US	1993–2015	ER (8), SR (28)	36	5	5
Vanoli et al.	2017	Italy	1980–2015	ER (56), SR (147)^c^	175	NA	52
Masui et al.	2018	Japan	2000–2016	SR (31)	31	18	18
Zhang et al.	2019	US	1997–2016	ER (30), SR (131)^a^	162	61	61
Lee et al.	2019	Korea	2004–2017	ER (44), SR (16)^b^	59	0	0
Oono et al.	2019	Japan	2010–2018	ER (12)	12	NA	0
Fujimoto et al.	2019	Japan	2013–2017	ER (7), SR (3)^c^	10	0	0
Nießen et al.	2020	Germany	2002–2017	SR (22)	22	15	15
Exarchou et al.	2021	UK	2007–2020	ER (12), SR (5)	17	1	1
Matsueda et al.	2021	Japan	2005–2020	ER (34), SR (9)	43	3	3
Ragheb et al.	2021	US	2003–2018	ER (63)	63	NA	0
Tashima et al.	2021	Japan	2017–2020	ER (13)	13	NA	0
Inokuchi et al.	2022	Japan	2003–2020	ER (9), SR (2)	11	1	1
Nakao et al.	2022	Japan	2000–2020	ER (23), SR (32)	55	4	4
Ryu et al.	2022	Korea	2008–2020	ER (18), SR (11)	29	1	1

*ER* endoscopic resection, *LNM* lymph node metastasis, *NA* not applicable, *SR* surgical resection, *UK* United Kingdom, *US* United States

^a^Including some cases with undetailed resection methods; six cases in the report by Rosentraeger et al. and one case in the report by Zhang et al.

^b^Including 12 cases with additional gastrectomy; three in the report by Gingul et al., six in the report by Lee et al., and three in the report by Fujimoto et al.

^c^Including 27 cases with neuroendocrine carcinomas, which were excluded from this meta-analysis

### Pooled prevalence of LNM in D-NETs

**Fig. 2:**
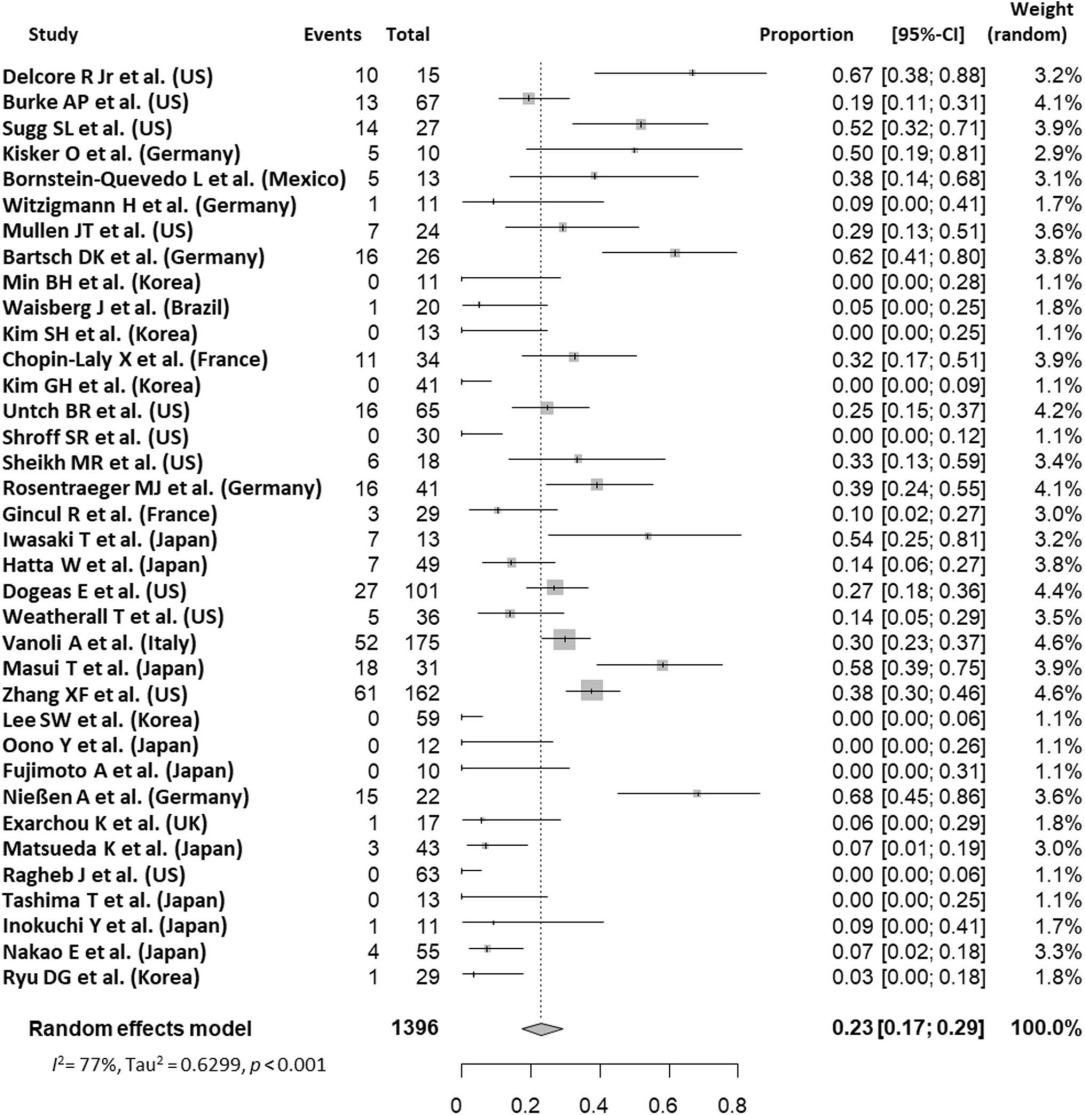
A forest plot of the meta-analysis of the prevalence of LNM in D-NETs. *CI* confidence interval, *D-NETs* duodenum neuroendocrine tumors, *LNM* lymph node metastasis, *UK* United Kingdom, *US* United States

Pooled data from all 36 studies that involved 1,396 patients with D-NETs identified 326 patients with LNM. The pooled prevalence of LNM in D-NETs was 22.7% (95% CI 17.3–29.2%), with considerable heterogeneity observed across the studies (*I*^2^ = 77%) (Fig. 2).

The pooled prevalences of LNM were 46.8% (95% CI 32.4–61.8%) for AD-NETs and 14.1% (95% CI 9.3–20.9%) for NAD-NETs when D-NETs were divided (Table 2). Further, the pooled prevalence of LNM for functional D-NETs was 53.3% (95% CI 45.9–60.6%). Substantial heterogeneity remained in NAD-NETs analyses despite the absence of significant heterogeneity in the analysis of AD-NETs and functional D-NETs (*I*^2^ = 73.8%). When cases were limited to non-functional NAD-NETs, the heterogeneity decreased to moderate levels (*I*^2^ = 49.7%) compared with the overall NAD-NETs.

**Table 2:** Pooled prevalences in D-NETs according to tumor location and functionality

	No. of studies	No. of cases	No. of LNM	Pooled prevalence, % (95% CI)		*p* for *Q* test	*I*^2^	*p* for Egger’s test^a^
Overall	36	1396	326	22.7	(17.3–29.2)	< 0.001	77.4	0.002
Tumor location								
AD-NETs	6	80	36	46.8	(32.4–61.8)	0.256	23.7	–
NAD-NETs	25	851	141	14.1	(9.3–20.9)	< 0.001	73.8	< 0.001
Tumor functionality								
Functional D-NETs	8	181	96	53.3	(45.9–60.6)	0.540	0.0	–
Non-functional AD-NETs	4	40	18	49.2	(25.0–73.8)	0.160	41.9	–
Non-functional NAD-NETs	20	607	50	9.5	(6.1–14.7)	0.006	49.7	< 0.001

*AD-NETs* ampullary/peri-ampullary duodenal neuroendocrine tumor, *CI* confidence interval, *D-NETs* duodenal neuroendocrine tumors, *LNM* lymph node metastasis, *NAD-NETs* non-ampullary duodenal neuroendocrine tumors

^a^Egger’s test was not performed in the analysis due to the number of studies being < 10

### Subgroup analyses for exploring the heterogeneity of NAD-NETs

A NAD-NETs subgroup analysis revealed no significant differences in the prevalence of LNM among the geographic areas (*p*_interaction_ = 0.451) (Table 3). Conversely, a significant difference was observed among the different treatment methods (*p*_interaction_ < 0.001). The analysis limited to non-functional NAD-NETs, demonstrated comparable results.

**Table 3:** Subgroup analyses in the pooled prevalences of LNM in NAD-NETs

	No. of studies	No. of cases	No. of LNM	Pooled prevalence, % (95% CI)		*p*_interaction_
Geographic area						
NAD-NETs						0.451
Asia	12	343	35	8.7	(3.4–20.8)	
Europe	5	120	31	23.2	(12.3–39.4)	
North America	7	368	74	18.1	(10.1–30.3)	
Non-functional NAD-NETs						0.375
Asia	10	295	15	7.4	(3.7–14.4)	
Europe	4	59	5	9.9	(4.3–21.0)	
North America	5	233	29	14.0	(6.4–28.0)	
Treatment method						
NAD-NETs						< 0.001
ER	20	423	2	3.1	(1.7–5.6)	
SR	19	363	128	33.6	(25.0–43.5)	
Non-functional NAD-NETs						< 0.001
ER	18	374	1	3.2	(1.7–5.6)	
SR	14	186	44	25.9	(17.9–36.0)	

*CI* confidence interval, *ER* endoscopic resection, *LNM* lymph node metastasis, *NAD-NETs* non-ampullary duodenal neuroendocrine tumors, *SR* surgical resection

### Pathological risk factors of LNM for NAD-NETs

Regarding pathological risk factors for LNM in NAD-NETs, tumor size > 10 mm (OR, 7.31; 95% CI 3.28–16.31), tumor invasion into the MP or deeper (OR, 7.79; 95% CI 3.65–16.61), WHO grading of G2 (OR, 2.47; 95% CI 1.03–5.92), and the presence of LVI (OR, 5.67; 95% CI 2.29–14.06) were significantly associated with LNM (Table 4). The analysis for overall D-NETs revealed comparable results (Supplementary Table 3; Supplementary Fig. 2). The WHO grading of G3 could not be analyzed because only one and seven cases were reported in NAD-NETs and overall D-NETs, respectively. The analysis for each pathological risk factor revealed no significant heterogeneity.

### Publication bias

Egger’s test indicated the potential publication biases in the analysis of the prevalences for overall D-NETs, NAD-NETs, and non-functional NAD-NETs (Table 2). Further, the visual assessment of the funnel plots (Supplementary Fig. 3) confirmed these biases. In contrast, the analyses of pathological risk factors for LNM demonstrated no publication bias (Table 4).

**Table 4:** Pooled ORs for the pathological risk factors of LNM in NAD-NETs

	No. of tudies	No. of cases	No. of LNM	Pooled OR, (95% CI)	*p*	*p* for Q test	*I*^2^	*p* for Egger’s test^a^
Tumor size								
≤ 10 mm		246	13	Reference				
> 10 mm	14	77	25	7.31 (3.28–16.31)	< 0.001	0.880	0.0	0.909
Tumor depth								
Mucosa/SM		277	15	Reference				
MP or deeper	15	52	26	7.79 (3.65–16.61)	< 0.001	0.775	0.0	0.108
WHO grading								
G1		181	27	Reference				
G2	9	30	9	2.47 (1.03–5.92)	0.043	0.709	0.0	–
LVI								
Negative		219	12	Reference				
Positive	12	47	14	5.67 (2.29–14.06)	< 0.001	0.837	0.0	0.923

*CI* confidence interval, *LNM* lymph node metastasis, *LVI* lymphovascular invasion, *MP* muscularis propria, *NAD-NETs* non-ampullary duodenal neuroendocrine tumors, *OR* odds ratio, *SM* submucosa, *WHO* World Health Organization

^a^Egger’s test was used, if there were ≥ 10 studies in the meta-analysis

## Discussion

Although ER has been accepted for small D-NETs [57], particularly for non-functional NAD-NETs [51], knowledge about the potential LNM risk remains limited. This systematic review revealed not only the prevalence of LNM in overall D-NETs (22.7%) but also the differences in the prevalences of LNM across tumor locations (AD-NETs and NAD-NETs) and functionality (functional and non-functional NAD-NETs). Additionally, tumor size > 10 mm, tumor invasion into the MP or deeper, WHO grading of G2, and the presence of LVI were identified as the pathological risk factors for LNM in non-functional NAD-NETs.

This systematic review highlighted two key clinical implications for deciding the treatment strategy based on the risk of LNM. First, the prevalence of LNM in each D-NET was different according to the tumor location; the high and low rates in AD-NETs (46.8%) and NAD-NETs (14.1%), respectively. Further, tumor functionality affected the prevalence of LNM, particularly functional D-NETs demonstrated a high rate at 53.3%, whereas non-functional NAD-NETs exhibited a low rate at 9.5%. These results could support the current European Neuroendocrine Tumor Society (ENETS) and National Comprehensive Cancer Network (NCCN) guidelines, which recommend tailoring treatment strategies based on tumor location and functionality [10, 11]. These guidelines recommended ER, despite very low-grade evidence, primarily for non-functional NAD-NETs, whereas SR is recommended for AD-NETs and functional D-NETs due to the higher risk of LNM. Although this scarcity of robust data prompts caution regarding the applicability and reliability of these recommendations, our results emphasized the differences in clinical aggressiveness among D-NETs and indicated that ER could be primarily considered only in cases with NAD-NETs.

Second, this study elucidated the pathological risk factors of LNM for NAD-NETs. While the NCCN guideline provides no specific details on the pathological features for considering SR [11], the ENETS guideline recommends that SR may be indicated in D-NETs with a tumor size > 10 mm, tumor invading the muscularis layer, WHO grading of G2/G3, and the presence of LVI, but it is clearly stated that this recommendation is based on very low-grade evidence [10]. Our findings indicated that tumor size > 10 mm (OR, 7.31), tumor invasion into the MP or deeper (OR, 7.79), WHO grading of G2 (OR, 2.47), and the presence of LVI (OR, 5.67) were significantly associated with LNM. Our results support the guidelines’ recommendations, but some cases still develop LNM without these risk factors. Specifically, the prevalence of LNM in grading G1 NETs was unexpectedly high (27 of 181 cases), which might explain the relatively lower OR for WHO grading of G2. Confounding factors, such as G1 NETs of > 10 mm, invasion into the MP, or positive LVI, could have affected these findings. Although we cannot completely rule out the possibility of LNM in cases without these pathological risk factors, these findings, in addition to the tumor location and functionality, could be crucial in determining the indications for ER and SR in D-NETs.

The cause of the heterogeneity observed in each analysis needs to be discussed. In the analysis of overall D-NETs, observing heterogeneity was expected due to the varying aggressiveness of each D-NET type, as previously mentioned. As a result, the heterogeneities decreased when analyses were performed according to each specific type. The subgroup analysis implied the potential effect of the treatment method (*p*_interaction_ < 0.001), although moderate heterogeneity persisted in the analysis of non-functional NAD-NETs. It can be assumed that the pathological features were different between cases treated with ER and SR, as ER is generally selected for smaller and less invasive tumors. Further, these pathological features might greatly affect LNM as the type of D-NETs, as shown in the analysis of the pathological risk factor. Moreover, the lower LNM rate in ER cases compared with SR cases may not reflect the actual prevalence, as radiological assessment of LNM with a short follow-up duration in ER cases may result in false-negative cases. These might explain the cause of the heterogeneity observed among non-functional NAD-NETs.

This systematic review focused on the risk of LNM in D-NETs, as the selection of treatment methods, such as ER, SR, and additional SR after ER, was recommended based on the presence or absence of LNM in the guidelines [10]. However, the impact of LNM on prognosis remains controversial. A study demonstrated that LNM was associated with poor disease-free survival after SR, although this study included both D-NETs and duodenal neuroendocrine carcinomas [45]. Conversely, other reports did not show an association between LNM and prognosis [42, 46]. Furthermore, the role of LNM in prognosis for cases treated with ER or local resection remains unclear. A large-scale study that includes ER cases with a very long follow-up duration is needed to address this issue.

Our study has several strengths. First, this systematic review included the methodological rigor based on the PRISMA statement, the comprehensive literature search with well-defined inclusion criteria, careful exclusion of redundant studies, the inclusion of high-quality studies with detailed data extraction, rigorous assessment of study quality, and robust statistical methods to establish and/or refute the validity of the results of our meta-analysis. Second, to the best of our knowledge, this is the first systematic review and meta-analysis that specifically focused on LNM in D-NETs, including the largest number of cases. Third, despite our strict selection criteria, we included a relatively large number of studies, which enabled different subgroup analyses and exploration of the causes of heterogeneities.

This study has several limitations to be acknowledged. First, considerable heterogeneity was observed in the analysis of the prevalence of overall D-NETs. However, as previously mentioned, the inevitable inherent variability among the types of D-NETs partially explained this heterogeneity. Second, most studies included in our analysis were retrospective, which may introduce biases related to data collection and reporting. Third, LNM was confirmed clinically rather than pathologically in cases treated with ER or local resection. This study included ER cases with a short follow-up duration, which could lead to an underestimation of LNM in such cases. Indeed, the median and mean follow-up durations in reports including ER cases ranged from 12 to 108 months and 17 to 68 months, respectively. Thus, caution is required when interpreting LNM in ER cases, and as mentioned earlier, a large-scale study with a very long follow-up duration, is needed to address this limitation. Finally, pathological risk factors were assessed using univariate analysis because all included studies provided only univariate pathological information. Multivariate analysis is necessary to determine whether the risk factors identified in this systematic review are independent predictors of LNM in NAD-NETs or all D-NETs.

In conclusion, approximately one-fourth of the patients with D-NETs had LNM. The prevalence of LNM differed according to the location and functionality, in particular, high in AD-NETs and functional D-NETs, whereas low in non-functional NAD-NETs. In addition, a tumor size of > 10 mm, tumor invasion into the MP or deeper, WHO grading of G2, and the presence of LVI were pathological risk factors for LNM in NAD-NETs. ER might be acceptable for non-functional NAD-NETs with diameters of ≤ 10 mm, but additional SR with lymphadenectomy may be recommended for cases exhibiting MP invasion, WHO grading of G2, or the presence of LVI.

## Supplementary Information

Below is the link to the electronic supplementary material.

Supplementary file1 (DOCX 300 KB)

## Abbreviations

AD-NETs: Ampullary/peri-ampullary duodenal neuroendocrine tumors
CI: Confidence interval
D-NETs: Duodenal neuroendocrine tumors
ENETS: European Neuroendocrine Tumor Society
ER: Endoscopic resection
JBI: Joanna Briggs Institute
LNM: Lymph node metastasis
LVI: Lymphovascular invasion
MP: Muscularis propria
NAD-NETs: Non-ampullary duodenal neuroendocrine tumors
NCCN: National Comprehensive Cancer Network
NETs: Neuroendocrine tumors
OR: Odds ratio
PRISMA: Preferred Reporting Items for Systematic Reviews and Meta-Analyses
SR: Surgical resection
WHO: World Health Organization
