# Supplementary material for: Prevalence and risk factors for lymph node metastasis in duodenal neuroendocrine tumors: a systematic review and meta-analysis
Source: J Gastroenterol. 2025 Apr 3;60(6):673–82. doi: 10.1007/s00535-025-02247-7 (PMC12095403; doi:10.1007/s00535-025-02247-7)
Supplement: Supplementary file 1 — Supplementary file1 (DOCX 300 KB) [file 535_2025_2247_MOESM1_ESM.docx]

**Supplementary files**

**Supplementary Appendix 1.** PRISMA 2020 Main Checklist

| **Topic** | **No.** | **Item** | **Location where item is reported** |
| --- | --- | --- | --- |
| **TITLE** |  |  |  |
| **Title** | 1 | Identify the report as a systematic review. | p1 |
| **ABSTRACT** |  |  |  |
| **Abstract** | 2 | See the PRISMA 2020 for Abstracts checklist | p3,4 |
| **INTRODUCTION** |  |  |  |
| **Rationale** | 3 | Describe the rationale for the review in the context of existing knowledge. | p6,7 |
| **Objectives** | 4 | Provide an explicit statement of the objective(s) or question(s) the review addresses. | p7 |
| **METHODS** |  |  |  |
| **Eligibility criteria** | 5 | Specify the inclusion and exclusion criteria for the review and how studies were grouped for the syntheses. | p8,9 |
| **Information sources** | 6 | Specify all databases, registers, websites, organisations, reference lists and other sources searched or consulted to identify studies. Specify the date when each source was last searched or consulted. | p8 |
| **Search strategy** | 7 | Present the full search strategies for all databases, registers and websites, including any filters and limits used. | Supplementary  Appendix 2 |
| **Selection process** | 8 | Specify the methods used to decide whether a study met the inclusion criteria of the review, including how many reviewers screened each record and each report retrieved, whether they worked independently, and if applicable, details of automation tools used in the process. | P8,9,10 |
| **Data collection process** | 9 | Specify the methods used to collect data from reports, including how many reviewers collected data from each report, whether they worked independently, any processes for obtaining or confirming data from study investigators, and if applicable, details of automation tools used in the process. | p10 |
| **Data items** | 10a | List and define all outcomes for which data were sought. Specify whether all results that were compatible with each outcome domain in each study were sought (e.g. for all measures, time points, analyses), and if not, the methods used to decide which results to collect. | p9,10 |
|  | 10b | List and define all other variables for which data were sought (e.g. participant and intervention characteristics, funding sources). Describe any assumptions made about any missing or unclear information. | p10 |
| **Study risk of bias assessment** | 11 | Specify the methods used to assess risk of bias in the included studies, including details of the tool(s) used, how many reviewers assessed each study and whether they worked independently, and if applicable, details of automation tools used in the process. | p11,12,13 |
| **Effect measures** | 12 | Specify for each outcome the effect measure(s) (e.g. risk ratio, mean difference) used in the synthesis or presentation of results. | p11,12 |
| **Synthesis methods** | 13a | Describe the processes used to decide which studies were eligible for each synthesis (e.g. tabulating the study intervention characteristics and comparing against the planned groups for each synthesis (item 5)). | p11,12 |
|  | 13b | Describe any methods required to prepare the data for presentation or synthesis, such as handling of missing summary statistics, or data conversions. | p11,12 |
|  | 13c | Describe any methods used to tabulate or visually display results of individual studies and syntheses. | p11,12 |
|  | 13d | Describe any methods used to synthesize results and provide a rationale for the choice(s). If meta-analysis was performed, describe the model(s), method(s) to identify the presence and extent of statistical heterogeneity, and software package(s) used. | p11,12 |
|  | 13e | Describe any methods used to explore possible causes of heterogeneity among study results (e.g. subgroup analysis, meta-regression). | p11,12 |
|  | 13f | Describe any sensitivity analyses conducted to assess robustness of the synthesized results. | p11,12 |
| **Reporting bias assessment** | 14 | Describe any methods used to assess risk of bias due to missing results in a synthesis (arising from reporting biases). | p11,12 |
| **Certainty assessment** | 15 | Describe any methods used to assess certainty (or confidence) in the body of evidence for an outcome. | p11,12 |
| **RESULTS** |  |  |  |
| **Study selection** | 16a | Describe the results of the search and selection process, from the number of records identified in the search to the number of studies included in the review, ideally using a flow diagram. | p14, Fig1 |
|  | 16b | Cite studies that might appear to meet the inclusion criteria, but which were excluded, and explain why they were excluded. | Fig1 |
| **Study characteristics** | 17 | Cite each included study and present its characteristics. | Table1, S1 |
| **Risk of bias in studies** | 18 | Present assessments of risk of bias for each included study. | Table S2 |
| **Results of individual studies** | 19 | For all outcomes, present, for each study: (a) summary statistics for each group (where appropriate) and (b) an effect estimate and its precision (e.g. confidence/credible interval), ideally using structured tables or plots. | p15,16,  Fig2,  Table2 |
| **Results of syntheses** | 20a | For each synthesis, briefly summarise the characteristics and risk of bias among contributing studies. | p15,16 |
|  | 20b | Present results of all statistical syntheses conducted. If meta-analysis was done, present for each the summary estimate and its precision (e.g. confidence/credible interval) and measures of statistical heterogeneity. If comparing groups, describe the direction of the effect. | p15,16 |
|  | 20c | Present results of all investigations of possible causes of heterogeneity among study results. | p15,16 |
|  | 20d | Present results of all sensitivity analyses conducted to assess the robustness of the synthesized results. | p15,16 |
| **Reporting biases** | 21 | Present assessments of risk of bias due to missing results (arising from reporting biases) for each synthesis assessed. | p16 |
| **Certainty of evidence** | 22 | Present assessments of certainty (or confidence) in the body of evidence for each outcome assessed. | p15,16 |
| **DISCUSSION** |  |  |  |
| **Discussion** | 23a | Provide a general interpretation of the results in the context of other evidence. | p17,18,19,  20,21 |
|  | 23b | Discuss any limitations of the evidence included in the review. | p20 |
|  | 23c | Discuss any limitations of the review processes used. | p20 |
|  | 23d | Discuss implications of the results for practice, policy, and future research. | p17,18,19,  20,21 |
| **OTHER INFORMATION** |  |  |  |
| **Registration and protocol** | 24a | Provide registration information for the review, including register name and registration number, or state that the review was not registered. | p8 |
|  | 24b | Indicate where the review protocol can be accessed, or state that a protocol was not prepared. | p8 |
|  | 24c | Describe and explain any amendments to information provided at registration or in the protocol. | p8 |
| **Support** | 25 | Describe sources of financial or non-financial support for the review, and the role of the funders or sponsors in the review. | p22 |
| **Competing interests** | 26 | Declare any competing interests of review authors. | p22 |
| **Availability of data, code and other materials** | 27 | Report which of the following are publicly available and where they can be found: template data collection forms; data extracted from included studies; data used for all analyses; analytic code; any other materials used in the review. | Table1  TableS1 |

*From:* Page MJ, McKenzie JE, Bossuyt PM, Boutron I, Hoffmann TC, Mulrow CD, et al. The PRISMA 2020 statement: an updated guideline for reporting systematic reviews. MetaArXiv. 2020, September 14. DOI: 10.31222/osf.io/v7gm2. For more information, visit: [www.prisma-statement.org](file:///C:\Users\yyuan\Dropbox\001-Home%20office%20back%20up\Takeshi-prevalence%20and%20risk%20factors%20for%20LNM%20in%20D-NETs\www.prisma-statement.org)

**Supplementary Appendix 2.** Literature research

**Databases:**

EBM Reviews - Cochrane Central Register of Controlled Trials <May 2023>, EBM Reviews - Cochrane Database of Systematic Reviews <2005 to June 6, 2023>, Embase <1974 to 2023 June 09>, OVID Medline Epub Ahead of Print, In-Process & Other Non-Indexed Citations, Ovid MEDLINE(R) Daily and Ovid MEDLINE(R) 1946 to Present

**Search Strategy:**
**1**  exp Neuroendocrine Tumors/ or exp neuroendocrine tumor/ (373231)
**2**  (neuroendocrine adj3 (tumor* or tumour* or carcinoma* or cancer* or malignan* or neoplas*)).tw,kw. (78201)
**3**  carcinoid*.tw,kw. (40348)
**4**  (APUD adj3 (tumor* or tumour* or carcinoma* or cancer* or malignan* or neoplas*)).tw,kw. (259)
**5**  (NETs or Mixed adenoneuroendocrine carcinoma* or MANEC or apudoma*).tw,kw. (44472)
**6**  or/1-5 (433843)
**7**  exp Gastrointestinal Tract/ (786704)
**8**  (stomach or gastric or gastro* or intragastric or duoden* or esophag* or oesophag* or intestin* or enteral or enteric or cecum or cecal or ileum or ileal or ileocecal or jejunum or jejunal).tw,kw. (3194159)
**9**  (colorectum or colon or rectum or rectal or small bowel or large bowel).tw,kw. (851140)
**10**  (GI tract or UGI tract or LGI tract or digestive tract or alimentary tract or Gut).tw,kw. (404644)
**11**  or/7-10 (4179498)
**12**  6 and 11 (55665)
**13**  exp Lymph Nodes/ (307156)
**14**  Lymph*.tw,kw. (2558263)
**15**  (LN or LNs).tw,kw. (76409)
**16**  exp neoplasm metastasis/ (1016512)
**17**  (metastasis or metastases).tw,kw. (1074906)
**18**  or/13-17 (3757450)
**19**  12 and 18 (21538)
**20**  conference abstract.pt. or Congresses as Topic/ or Conference Review.pt. (4927703)
**21**  19 not 20 (16875)
**22**  limit 21 to english language [Limit not valid in CDSR; records were retained] (14173)
**23**  (exp animals/ or exp animal/ or exp nonhuman/ or exp animal experiment/ or animal model/ or animal tissue/ or non human/) not (humans/ or human/ or human experiment/) (12551191)
**24**  22 not 23 use ppez,oemezd (13819)
**25**  (rat or rats or mice or mouse or swine or porcine or murine or sheep or lambs or pigs or piglets or rabbit or rabbits or cat or cats or dog or dogs or cattle or bovine or monkey or monkeys or trout or marmoset$1).ti. not (human* or men or women or patients or subjects or paticipants).tw. (3756471)
**26**  24 not 25 use ppez,oemezd (13807)
**27**  19 use coch,cctr (235)
**28**  26 or 27 (13813)

**29** limit 28 to yr="1990 -Current" (11212)

**30** remove duplicates from 29 (7769)

**Supplementary Tables**

**Supplementary Table 1.** Detailed characteristics of the included studies in this systematic review

| Authors  Year (Country) | Setting/  Study design | Enrollment  time period | Type of  treatment | No. of  patients | LNM in surgery | LNM in all cases | 2 x 2 table calculation for OR | | | | Location/  Functionality |
| --- | --- | --- | --- | --- | --- | --- | --- | --- | --- | --- | --- |
|  |  |  |  |  |  |  | Tumor size  > 10mm | Tumor depth  of MP or  deeper | LVI | G2/G3 |  |
| Delcore R Jr et al.  1990 (US) | Single center/  Retrospective cohort | 1960–1990 | SR (15) | 15 | 10 | 10 | NA | NA | NA | NA | NA/  All gastrinomas |
| Burke AP et al.  1990 (US) | Single center/  Retrospective cohort | 1970–1986 | SR (67) | 67 | 13 | 13 | NA | Available | NA | NA | NAD (52), AD (15)^¶^/  Functional NETs (12)^¶^ |
| Sugg SL et al.  1993 (US) | Single center/  Prospective cohort | 1989– | SR (27) | 27 | 14 | 14 | NA | NA | NA | NA | NA/  All gastrinomas |
| Kisker O et al.  1998 (Germany) | Single center/  Retrospective cohort | 1987–1996 | SR (10) | 10 | 5 | 5 | NA | NA | NA | NA | NA/  All gastrinomas |
| Bornstein-Quevedo L et al.  2001 (Mexico) | Single center/  Retrospective cohort | 1980–2000 | SR (13) | 13 | 5 | 5 | NA | NA | NA | NA | NAD (8), AD (5)/  Functional NETs (3)^¶^ |
| Witzigmann H et al.  2002 (Germany) | Multi center/  Retrospective cohort | 1992–2001 | ER (2),  SR (9) | 11 | 1 | 1 | Available | Available | NA | NA | NAD (6), AD (5)/  Functional NETs (7) |
| Mullen JT et al.  2005 (US) | Single center/  Retrospective cohort | 1969–2004 | ER (6),  SR (18) | 24 | 7 | 7 | Available | Available | Available | NA | All NAD/  Functional NETs (0) |
| Bartsch DK et al.  2012 (Germany) | Multi center/  Retrospective cohort | 1990–2011 | SR (26) | 26 | 16 | 16 | NA | NA | NA | NA | NA/  All gastrinomas |
| Min BH et al.  2013 (Korea) | Single center/  Retrospective cohort | 1996–2009 | ER (11) | 11 | NA | 0 | Available | Available | Available | NA | All NAD/  Functional NETs (0) |
| Waisberg J et al.  2013 (Brazil) | Single center/  Retrospective cohort | 1993–2011 | ER (15),  SR (5) | 20 | 1 | 1 | NA | NA | NA | NA | All NAD/  Functional NETs (0) |
| Kim SH et al.  2013 (Korea) | Single center/  Retrospective cohort | 2001–2011 | ER (12),  SR (1) | 13 | 0 | 0 | Available | Available | Available | Available | All NAD/  Functional NETs (1)^¶^ |
| Chopin-Laly X et al.  2013 (France) | Single center/  Retrospective cohort | 1991–2007 | ER (NA),  SR (NA) | 34 | NA | 11 | NA | NA | NA | NA | All NAD/  Functional NETs (6)^¶^ |
| Kim GH et al.  2014 (Korea) | Multi center/  Retrospective cohort | 2006–2011 | ER (41) | 41 | NA | 0 | Available | Available | Available | NA | All NAD/  Functional NETs (0) |
| Untch BR et al.  2014 (US) | Single center/  Retrospective cohort | 1983–2011 | ER (12),  SR (53) | 65 | 16 | 16 | NA | NA | NA | Available | NAD (50), AD (23)^†§¶^  Functional NETs (7)^¶^ |
| Shroff SR et al.  2015 (US) | Single center/  Retrospective cohort | 2001–2011 | ER (20),  SR (10) | 30 | 0 | 0 | Available | Available | Available | NA | All NAD/  NA |
| Sheikh MR et al.  2016 (US) | Single center/  Retrospective cohort | 2005–2014 | SR (18) | 18 | 7 | 6 | Available | Available | NA | NA | NA/  NA |
| Rosentraeger MJ et al.  2016 (Germany) | Single center/  Retrospective cohort | 1984–2008 | ER (10),  SR (25)^§^ | 41 | 16 | 16 | Available | Available | NA | Available | Non functional NAD (17),  Functional NETs (24) |
| Gincul R et al.  2016 (France) | Multi center/  Retrospective cohort | 1996–2003 | ER (26),  SR (3)^‡^ | 29 | 2 | 3 | Available | NA | NA | NA | NAD (22), AD (7)/  NA |
| Iwasaki T et al.  2017 (Japan) | Single center/  Retrospective cohort | 2000–2015 | SR (13) | 13 | 7 | 7 | Available | Available | Available | Available | NAD (6), AD (7)/  Functional NETs (0) |
| Hatta W et al.  2017 (Japan) | Multi center/  Retrospective cohort | 1992–2013 | ER (35),  SR (14) | 49 | 6 | 7 | Available | Available | Available | Available | All NAD/  Functional NETs (4) |
| Dogeas E et al.  2017 (US) | Single center/  Retrospective cohort | 1996–2012 | ER (38),  SR (63) | 101 | 27 | 27 | Available | NA | NA | NA | NAD(80), AD(21)/  Functional NETs (0) |
| Weatherall T et al.  2017 (US) | Single center/  Retrospective cohort | 1993–2015 | ER (8),  SR (28) | 36 | 5 | 5 | NA | Available | Available | Available | All NAD/  Functional NETs (0) |
| Vanoli A et al.  2017 (Italy) | Multi center/  Retrospective cohort | 1980–2015 | ER (56),  SR (147)^†^ | 175 | NA | 52 | NA | Available | Available | Available | NAD (119), AD (55)^§¶^/  Functional NETs (69) |
| Masui T et al.  2018 (Japan) | Single center/  Retrospective cohort | 2000–2016 | SR (31) | 31 | 18 | 18 | NA | NA | NA | NA | All NAD/  Functional NETs (19)^¶^ |
| Zhang XF et al.  2019 (US) | Multi center/  Retrospective cohort | 1997–2016 | ER (30),  SR (131)^§^ | 162 | 61 | 61 | Available | NA | NA | NA | NAD (127), AD (35)/  Functional NETs (34)^¶^ |
| Lee SW et al.  2019 (Korea) | Multi center/  Retrospective cohort | 2004–2017 | ER (44),  SR (16)^‡^ | 59 | 0 | 0 | NA | NA | NA | NA | All NAD/  NA |
| Oono Y et al.  2019 (Japan) | Single center/  Retrospective cohort | 2010–2018 | ER (12) | 12 | NA | 0 | Available | Available | NA | Available | All NAD/  NA |
| Fujimoto A et al.  2019 (Japan) | Single center/  Retrospective cohort | 2013–2017 | ER (7),  SR (3)^‡^ | 10 | 0 | 0 | Available | Available | Available | NA | All NAD/  NA |
| Nießen A et al.  2020 (Germany) | Single center/  Retrospective cohort | 2002–2017 | SR (22) | 22 | 15 | 15 | Available | NA | Available | Available | NA/  Functional NETs (0) |
| Exarchou K et al.  2021 (UK) | Single center/  Prospective cohort | 2007–2020 | ER (12),  SR (5) | 17 | 1 | 1 | NA | NA | NA | NA | All NAD/  Functional NETs (0) |
| Matsueda K et al.  2021 (Japan) | Single center/  Retrospective cohort | 2005–2020 | ER (34),  SR (9) | 43 | 3 | 3 | Available | Available | Available | Available | All NAD/  Functional NETs (0) |
| Ragheb J et al.  2021 (US) | Multi center/  Retrospective cohort | 2003–2018 | ER (63) | 63 | NA | 0 | NA | NA | NA | NA | All NAD/  Functional NETs (0) |
| Tashima T et al.  2021 (Japan) | Single center/  Retrospective cohort | 2017–2020 | ER (13) | 13 | NA | 0 | NA | Available | Available | Available | All NAD/  NA |
| Inokuchi Y et al.  2022 (Japan) | Single center/  Retrospective cohort | 2003–2020 | ER (9),  SR (2) | 11 | 1 | 1 | NA | Available | Available | Available | NA/  NA |
| Nakao E et al.  2022 (Japan) | Single center/  Retrospective cohort | 2000–2020 | ER (23),  SR (32) | 55 | 4 | 4 | Available | Available | Available | Available | All NAD/  Functional NETs (0) |
| Ryu DG et al.  2022 (Korea) | Single center/  Retrospective cohort | 2008–2020 | ER (18),  SR (11) | 29 | 1 | 1 | NA | Available | Available | Available | NA/  Functional NETs (0) |

† Including some cases with neuroendocrine carcinomas in the presented number, which were excluded from this meta-analysis; 10 cases in the report by Untch et al. and 27 cases in the report by Vanoli et al.

‡ Including 12 cases with additional gastrectomy; three in the report by Gingul et al., six in the report by Lee et al., and three in the report by Fujimoto et al.

§ Including some undetailed cases; two cases of location in the report by Untch et al., six cases of treatment in the report by Rosentraeger et al., one case of location in the report by Vanoli er al., one case of treatment in the report by Zhang et al.

¶ Unavailability of the analysis divided into location or functionality.

AD, ampullary/periampullary duodenum; ER, endoscopic resection; LNM, lymph node metastasis; LVI, lymphovascular invasion; MP, muscularis propria; NA, not appliable; NAD, non-ampullary duodenum; NETs, neuroendocrine tumors; OR, odds ratio; SR, surgical resection; UK, United Kingdom; US, United States; WHO, World Health Organization.

**Supplementary Table 2.** Quality assessment of the included studies using the JBI Critical Appraisal Tools for JBI Systematic Reviews.

|  | 1. Was the sample frame appropriate to address the target population? | 2. Were study participants sampled in an appropriate way? | 3. Was the sample size adequate? | 4. Were the study subjects and the setting described in detail? | 5. Was the data analysis conducted with sufficient coverage of the identified sample? | 6. Were valid methods used for the identification of the condition? | 7. Was the condition measured in a standard, reliable way for all participants? | 8. Was there appropriate statistical analysis? | 9. Was the response rate adequate, and if not, was the low response rate managed appropriately? | Risk of bias | Overall appraisal |
| --- | --- | --- | --- | --- | --- | --- | --- | --- | --- | --- | --- |
| Delcore R Jr et al.  1990 (USA) | Yes | Yes | NA | Yes | Yes | Yes | Yes | Yes | NA | Low | Include |
| Burke AP et al.  1990 (USA) | Yes | Yes | NA | No | Yes | Yes | Yes | Yes | NA | Moderate | Include |
| Sugg SL et al.  1993 (USA) | Yes | Yes | NA | Yes | Yes | Yes | Yes | Yes | NA | Low | Include |
| Kisker O et al.  1998 (Germany) | Yes | Yes | NA | Yes | Yes | Yes | Yes | Yes | NA | Low | Include |
| Bornstein-Quevedo L et al.  2001 (Mexico) | Yes | Yes | NA | Yes | Yes | Yes | Yes | Yes | NA | Low | Include |
| Witzigmann H et al.  2002 (Germany) | Yes | Yes | NA | Yes | Yes | Yes | Yes | Yes | NA | Low | Include |
| Mullen JT et al.  2005 (USA) | Yes | Yes | NA | Yes | Yes | Yes | Yes | Yes | NA | Low | Include |
| Bartsch DK et al.  2012 (Germany) | Yes | Yes | NA | Yes | Yes | Yes | Yes | Yes | NA | Low | Include |
| Min BH et al.  2013 (Korea) | Yes | Yes | NA | Yes | Yes | Yes | Yes | Yes | NA | Low | Include |
| Waisberg J et al.  2013 (Brazil) | Yes | Yes | NA | Yes | Yes | Yes | Yes | Yes | NA | Low | Include |
| Kim SH et al.  2013 (Korea) | Yes | Yes | NA | Yes | Yes | No | Yes | Yes | NA | Moderate | Include |
| Chopin-Laly X et al.  2013 (France) | Yes | Yes | NA | Yes | Yes | Yes | Yes | Yes | NA | Low | Include |
| Kim GH et al.  2014 (Korea) | Yes | Yes | NA | Yes | Yes | No | Yes | Yes | NA | Moderate | Include |
| Untch BR et al.  2014 (USA) | Yes | Yes | NA | Yes | Yes | Yes | Yes | Yes | NA | Low | Include |
| Shroff SR et al.  2015 (USA) | Yes | Yes | NA | Yes | Yes | Yes | Yes | Yes | NA | Low | Include |
| Sheikh MR et al.  2016 (USA) | Yes | Yes | NA | No | Yes | Yes | Yes | Yes | NA | Moderate | Include |
| Rosentraeger MJ et al.  2016 (Germany) | Yes | Yes | NA | Yes | Yes | Yes | Yes | Yes | NA | Low | Include |
| Gincul R et al.  2016 (France) | Yes | Yes | NA | Yes | Yes | Yes | Yes | Yes | NA | Low | Include |
| Iwasaki T et al.  2017 (Japan) | Yes | Yes | NA | Yes | Yes | Yes | Yes | Yes | NA | Low | Include |
| Hatta W et al.  2017 (Japan) | Yes | Yes | NA | Yes | Yes | Yes | Yes | Yes | NA | Low | Include |
| Dogeas E et al.  2017 (USA) | Yes | Yes | NA | Yes | Yes | Yes | Yes | Yes | NA | Low | Include |
| Weatherall T et al.  2017 (USA) | Yes | Yes | NA | Yes | Yes | Yes | Yes | Yes | NA | Low | Include |
| Vanoli A et al.  2017 (Italy) | Yes | Yes | NA | Yes | Yes | Yes | Yes | Yes | NA | Low | Include |
| Masui T et al.  2018 (Japan) | Yes | Yes | NA | Yes | Yes | Yes | Yes | Yes | NA | Low | Include |
| Zhang XF et al.  2019 (USA) | Yes | Yes | NA | Yes | Yes | Yes | Yes | Yes | NA | Low | Include |
| Lee SW et al.  2019 (Korea) | Yes | Yes | NA | Yes | Yes | Yes | Yes | Yes | NA | Low | Include |
| Oono Y et al.  2019 (Japan) | Yes | Yes | NA | Yes | Yes | No | Yes | Yes | NA | Moderate | Include |
| Fujimoto A et al.  2019 (Japan) | Yes | Yes | NA | Yes | Yes | No | Yes | Yes | NA | Moderate | Include |
| Nießen A et al.  2020 (Germany) | Yes | Yes | NA | No | Yes | Yes | Yes | Yes | NA | Moderate | Include |
| Exarchou K et al.  2021 (UK) | Yes | Yes | NA | Yes | Yes | Yes | Yes | Yes | NA | Low | Include |
| Matsueda K et al.  2021 (Japan) | Yes | Yes | NA | Yes | Yes | Yes | Yes | Yes | NA | Low | Include |
| Ragheb J et al.  2021 (USA) | Yes | Yes | NA | Yes | Yes | Yes | Yes | Yes | NA | Low | Include |
| Tashima T et al.  2021 (Japan) | Yes | Yes | NA | Yes | Yes | No | Yes | Yes | NA | Moderate | Include |
| Inokuchi Y et al.  2022 (Japan) | Yes | Yes | NA | No | Yes | Yes | Yes | Yes | NA | Moderate | Include |
| Nakao E et al.  2022 (Japan) | Yes | Yes | NA | Yes | Yes | Yes | Yes | Yes | NA | Low | Include |
| Ryu DG et al.  2022 (Korea) | Yes | Yes | NA | No | Yes | Yes | Yes | Yes | NA | Moderate | Include |

JBI, Joanna Briggs Institute; NA, not applicable.

**Supplementary Table 3.** Pooled ORs for pathological risk factors of LNM in overall D-NETs.

|  |  | No. of  studies | No. of  cases | No. of  LNM | Pooled OR,  (95% CI) | *p* | *p* for Q test | *I^2^* | *p* for  Egger’s test^†^ |
| --- | --- | --- | --- | --- | --- | --- | --- | --- | --- |
| **Tumor size** | ≤ 10 mm |  | 301 | 29 | Reference |  |  |  |  |
|  | > 10 mm | 17 | 200 | 121 | 8.20 (4.43–15.15) | < 0.001 | 0.884 | 7.8 | 0.187 |
| **Tumor depth** | Mucosa/SM |  | 479 | 22^‡^ | Reference |  |  |  |  |
|  | MP or deeper | 20 | 174 | 47^‡^ | 6.75 (3.97–11.49) | < 0.001 | 0.790 | 0.0 | 0.113 |
| **WHO grading** | G1 |  | 402 | 49^‡^ | Reference |  |  |  |  |
|  | G2 | 14 | 86 | 23^‡^ | 3.86 (2.23–6.70) | < 0.001 | 0.913 | 0.0 | 0.914 |
| **LVI** | Negative |  | 383 | 22^‡^ | Reference |  |  |  |  |
|  | Positive | 16 | 125 | 25^‡^ | 6.97 (3.72–13.05) | < 0.001 | 0.881 | 0.0 | 0.730 |

† Egger’s test was used, if there were ≥ 10 studies in the meta-analysis.

‡ 52 cases with LNM reported by Vanoli, et al. (2017) were not included because whether these cases were classified into reference or risk factor was unclear. The results of logistic regression model in this study, not raw data, were pooled in this analysis.

CI, confidence interval; D-NETs, duodenal neuroendocrine tumors; LNM, lymph node metastasis; LVI, lymphovascular invasion; MP, muscularis propria; OR, odds ratio; SM, submucosa; WHO, World Health Organization.

**Supplementary Figures**

**
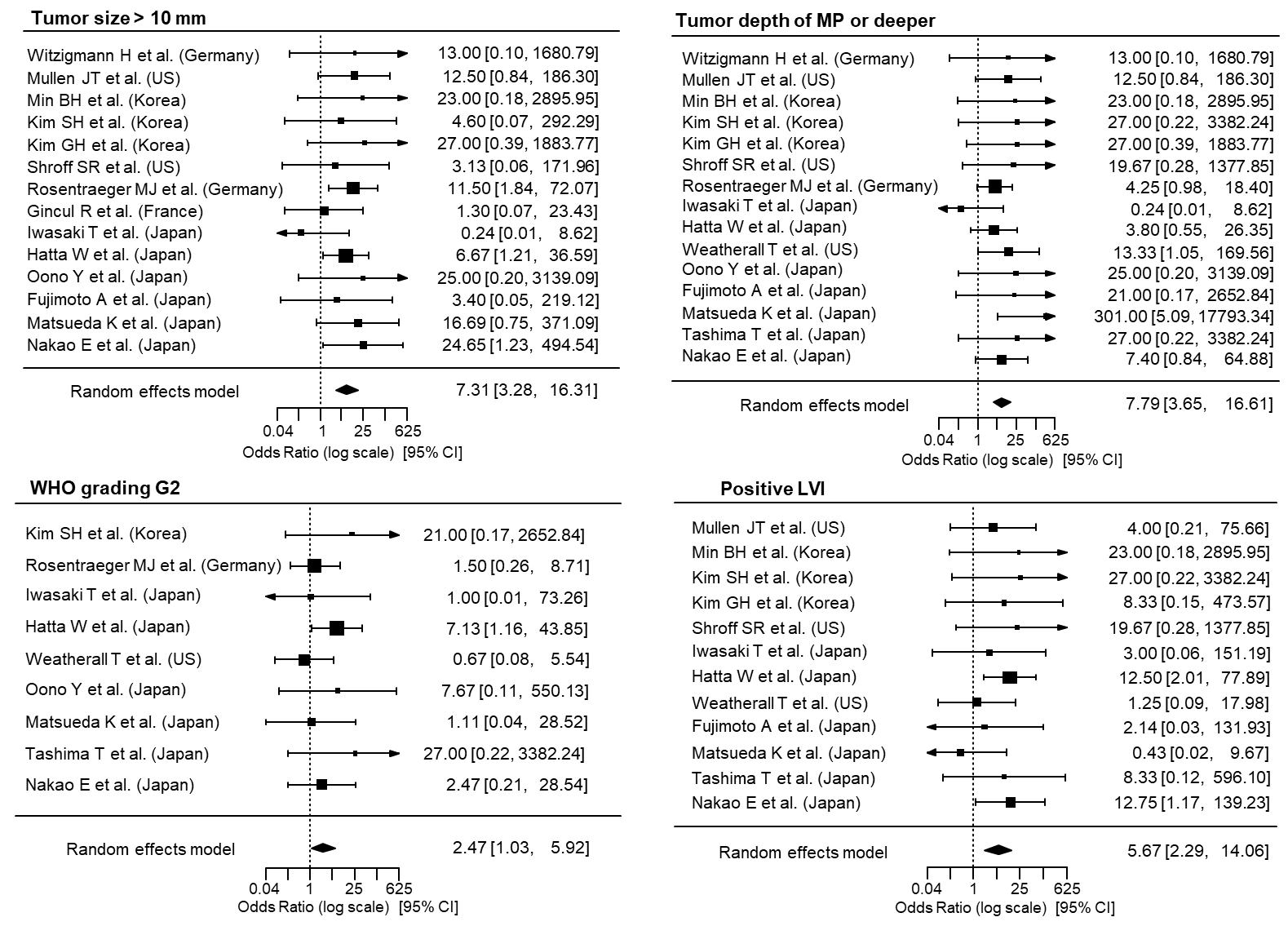
**

**Supplementary Fig. 1** Forest plots of odds ratios for pathological risk factors of LNM in NAD-NETs.

CI, confidence interval; LNM, lymph node metastasis; LVI, lymphovascular invasion; MP, muscularis propria; NAD-NETs, non-ampullary duodenal neuroendocrine tumors; SM, submucosa; US, United States; WHO, World Health Organization.

**
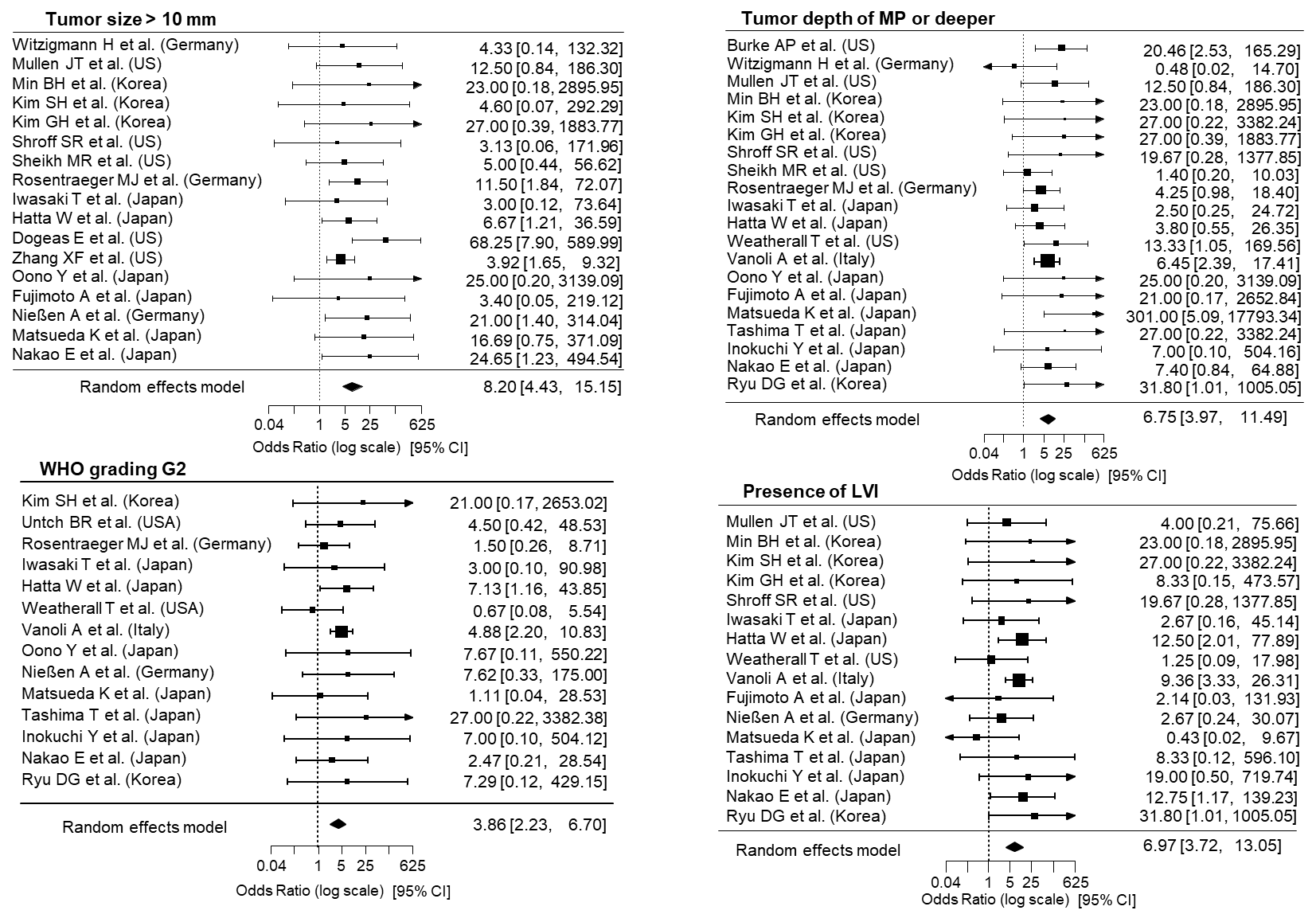
**

**Supplementary Fig. 2** Forest plots of odds ratios for pathological risk factors of LNM in overall D-NETs.

CI, confidence interval; D-NETs, duodenal neuroendocrine tumors; LNM, lymph node metastasis; LVI, lymphovascular invasion; MP, muscularis propria; SM, submucosa; US, United States; WHO, World Health Organization.

**
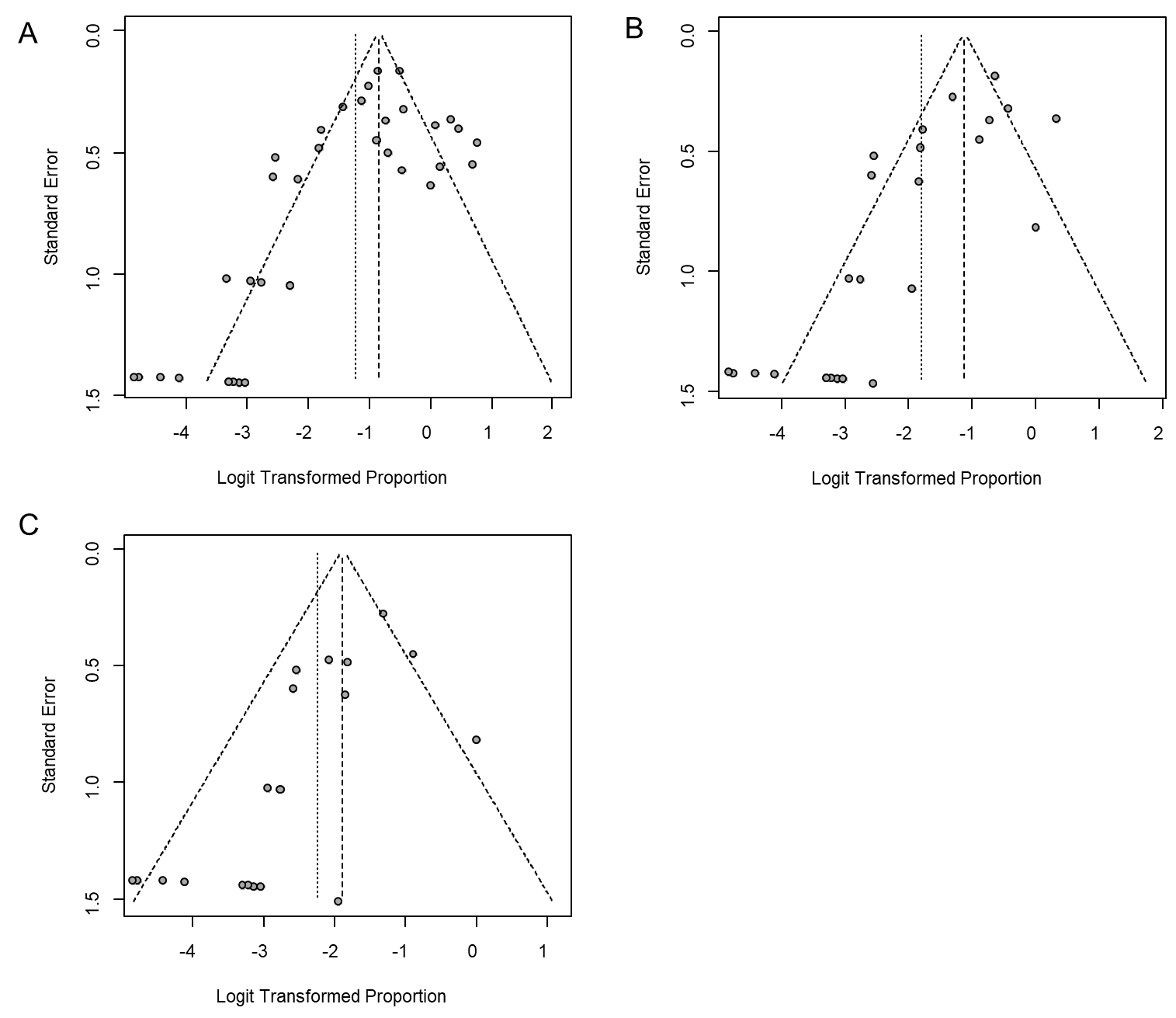
**

**Supplementary Fig. 3** Funnel plots for the analysis of the prevalence of LNM in overall D-NETs, NAD-NETs, and non-functional NAD-NETs. (A) Overall D-NETs; (B) NAD-NETs; and (C) non-functional NAD-NETs.

D-NETs, duodenal neuroendocrine tumors; LNM, lymph node metastasis; NAD-NETs, non-ampullary duodenal neuroendocrine tumors.
